# A realist synthesis of staff-based primary health care interventions addressing universal health coverage

**DOI:** 10.7189/jogh.12.04035

**Published:** 2022-05-14

**Authors:** Clelia D’Apice, Luca Ghirotto, Maria C Bassi, Giovanna Artioli, Leopoldo Sarli

**Affiliations:** 1 University of Parma, Department of Medicine and Surgery, Parma, Italy; 2 Qualitative Research Unit, Azienda USL – IRCCS, Reggio Emilia, Italy; 3 Medical Library, Azienda USL – IRCCS, Reggio Emilia, Italy

## Abstract

**Background:**

Universal Health Coverage (UHC) can be achieved by universal access to a solid and resilient people-centred health care system, with Primary Health Care (PHC) as its foundation and strategy. Increased access to PHC occurs when health care services are available, affordable, accessible, acceptable, and perceived appropriate by users. Many studies highlight that health care workers are critical in helping people access, navigate, and interact with PHC services. How the interventions involving health care staff work and under what circumstance remains unclear.

**Methods:**

Through a systematic review and a realist synthesis, we identified and described staff-based interventions impacting UHC through PHC. We conducted the systematic review from inception to June 2021, searching for peer-reviewed studies published in English, using quantitative methods for evaluating interventions.

**Results:**

We identified three Context-Mechanism-Outcome (CMO) configurations: inserting culturally sensitive ad hoc bridge figures, tailoring staff practices to the needs of specified populations, and training as a means for staff reskilling. Inserting ad hoc bridge figures in health care services was successful when they were familiar with the contextual culture and the users’ needs. The second configuration entails interventions where the staff was asked to consider the needs of targeted populations and differentiate strategies by the detected conditions. Finally, the third one consists of specific, ad hoc, and context-based training targeting several stakeholders. Central to this intervention was training for health care bridge figures, since they were explicitly trained before performing their duties to cope with the health care and social needs of the specific groups they intended to serve.

**Conclusions:**

The review highlights that the context and contextual factors should be considered for an intervention to be successful. Hence, it provides policymakers with practical indications for designing staff-based interventions for reaching UHC within PHC services in a given context. Healthcare bridge figures, an umbrella term embracing a variety of selected community health workers, often trained and working in the communities from which they come, increase access to PHC services as they respond to local societal and cultural norms and customs, ensuring community acceptance and ownership.

The United Nations has acknowledged Universal Health Coverage (UHC) as a critical objective [1]. Ensuring UHC means providing all people with access to high-quality health care services according to their needs, without financial hardship. Essential services range from health promotion to prevention, treatment, rehabilitation, and palliative care [2]. UHC is a critical approach that shifts the focus from disease-specific interventions to a people-centred, needs-related health care provision [3]. It is also a target at risk of being neglected, as the priority of the global health community changed because of the SARS-CoV-2 pandemic [4].

UHC does not always entail free access to health care and the form it takes depends on the country-specific health care systems, which may vary from one another by having either public, private, or mixed public/private health insurance systems. Nonetheless, the essence of UHC is the universal access to a solid and resilient people-centred health care system with Primary Health Care (PHC) as its foundation [5,6]. Addressing “access to health” and UHC requires identifying health care needs and determining the patients’ capabilities for seeking, obtaining, or using health care services, while also fulfilling the demand for the services [7]. This multi-dimension goal involves [8-10]:

- The services’ availability – the relation of the volume of existing health services and resources to the volume and type of patients' needs.
- Economic affordability – the relationship between the prices of services and patients' capability to pay.
- The services’ accessibility – the location of health services in relation to the location of patients (patients’ mobility).
- Their acceptability – cultural and social factors that affect an individual’s ability to accept or seek healthcare services.
- Accommodation – how the resources are organized to accept patients and patients’ perceptions of the appropriateness of these systems.

UHC is not only essential for affordable health care services and strengthening the health care system, but also for achieving equity in social care, public health, and health promotion [4]. Thus, PHC represents the first point of contact between the health care system and the population it intends to serve [11]. Conversely, UHC largely depends on a reliable PHC [12,13]. Improving UHC means solving problems related to the shortage of PHC. There is substantial evidence that enhanced or better access to PHC is associated with decreased emergency department utilization, decreased inpatient admissions, decreased surgeries, and lower costs [14,15]. Ensuring true UHC requires quality PHC designed around people [16].

PHC can be generally defined as a whole-of-society approach to health and well-being centred on the needs and preferences of individuals and communities. It addresses the broader determinants of health and focuses on the comprehensive and interrelated aspects of physical, mental, and social health and well-being, encompassing promotion, prevention, early intervention, treatment of acute conditions, vaccination, management of chronic diseases, and health education [17,18]. By providing care in and throughout a community, PHC addresses individual and family health needs, as well as the broader issues of public health and the needs of defined populations, qualifying for an potentially effective way to achieve health and health care for all [19-21]. PHC thus represents the essential framework to move towards UHC [22]. However, disparities in access to PHC still occur, and many people face barriers that decrease or impair their access to PHC services. Poor or limited access to PHC translates into disparities in health status and outcomes, increased hospitalization rates, augmented use of emergency departments, and reduced cost-effectiveness [23].

According to the definitions of access to UHC and provided PHC, likely improvements of UHC should be contextualized to differing perspectives, health needs, and material and cultural settings of diverse groups in a given society [24]. The context and contextual factors are recognized as critical elements to successful PHC delivery and implementation of health care interventions [25-28]: the theory underpinning our definition of PHC interventions considers them essential for the interventions’ success [29].

Given the growing number of studies on PHC interventions implemented worldwide [19,30,31], aggregating their evidence concerning contextual factors and comprehending their underlying functioning in terms of UHC is desirable. In the context of growing interest on UHC, recent reviews highlighted the importance of understanding the role and effectiveness of UHC interventions. For example, a scoping review identified facilitators and barriers to the use of implementation research in Africa [32]. Another review specified obstacles to access to care, while suggesting specific recommendations for further research [33]. Within this scenario, broadening the breadth of the review to a global level [4] would give an better overall perspective for health care providers and policymakers, since many reviews concentrated on Africa [32-34] or low-income areas or specific countries [35-37].

We selected the interventions involving staffing. There is substantial agreement on economic-related measures being fundamental for ensuring UHC within PHC [35,38-41]. However, while many studies highlight that health care workers are critical in helping people access, navigate, and interact with PHC services [23], how the interventions involving health care staff work and under what circumstances remains unclear.

## METHODS

By employing a systematic review, we aimed to retrieve the evidence from studies focusing on staff-based interventions in the context of PHC and UHC. We conducted a realist synthesis for identifying underlying reasons for the success/failure of the collected interventions.

A realist synthesis allows researchers to describe “what works under what circumstances” by disclosing specific interventions' interplay between context, mechanisms, and outcomes [42-44] of particular interventions.

By using a heuristic defined as the context-mechanism-outcome (CMO) configuration, realist synthesis develops evidence-informed theories about the interactions between intervention, mechanisms, and implementation contexts [44]. “Contexts” refer to the backdrop of programmes and research and are broadly understood as any condition that triggers and/or modifies the behaviour of a mechanism [29]. For this review, contexts also encompass whether the country of the intervention’s implementation is a low-, middle-, or high-income country, as well as the population characteristics (general population or vulnerable groups, such as minorities). Mechanisms are “underlying entities, processes, or structures which operate in particular contexts to generate outcomes of interest” [45]. Outcomes result from the interaction between a mechanism and its triggering context. For this review, outcomes were defined either as positive, if they increased access to PHC services, or negative, if they did not increase or unevenly increased access to PHC services.

The main review question was: Which staff-based interventions proved to impact UHC through PHC?

Related sub-questions were also asked:

- Which kinds of interventions are selected and implemented to increase access to PHC services?
- Which areas of intervention (treatment, follow-up, prevention including screening, general PHC) do they refer to?
- Do the interventions target a specific population group?
- Which is the role of contextual factors?

The study’s protocol was registered on PROSPERO on April 15, 2021, registration code: CRD42021232293. Reporting for this review followed the RAMESES publication standards for realist synthesis [46] and the PRISMA 2020 Statement [47].

### Inclusion and exclusion criteria

We included all the staff-based interventions targeting PHC access in the context of UHC.

“Access to PHC services” was understood as the opportunity to identify health care needs, seek, reach, obtain or use health care services, and fulfil the demand for services. “Intervention” was defined as all the strategies and actions involving specific professionals working for PHC providers for improving access to PHC services (please refer to search element 3 in **Table 1** for the complete list of professionals we included).

**Table 1:** Medline search strategy

Search element 1:	Universal health coverage; UHC; Health inequality*; Health equit*; Universal health insurance; Universal health care
**Search element 2:**	Primary health care; Primary care nursing; Physicians, primary care; Primary care
**Search element 3:**	Patient advocacy; Broker; Health broker; Health Service* broker; Community health worker; Community navigat*; Patient navigat*; Lay health work*; Link* to care; Navigat*; Lay worker*; Community health representative*; Community health advocate*; Cultur* broker; Link worker*; Liaison worker*; Care coordinator*; Indigenous health worker*; Patient advocat*; Lay navigator*; Health liaison*; Advocacy for health; Mediation; Enabling; Empowerment
**Search strategy**	universal health coverage[Title/Abstract] OR UHC[Title/Abstract] OR health inequalit*[Title/Abstract] OR health equit*[Title/Abstract] OR “Universal Health Insurance”[Mesh] OR “Universal Health Care”[Mesh]]
	**AND**
	(“Primary Health Care”[Mesh] OR “Primary Care Nursing”[Mesh] OR “Physicians, Primary Care”[Mesh]) OR (primary care[Title/Abstract])
	**AND**
	(“Patient Advocacy” [Mesh] OR broker* OR health broker* OR health service* broker OR community health worker* OR community navigat* OR peer navigat* OR patient navigat* OR lay health work* OR link* to care OR Navigat* OR lay worker* OR community health representative* OR community health advocate* OR cultur* broker OR link worker* OR liaison worker* OR care coordinator* OR indigenous health worker* OR patient advocat* OR lay navigator* OR health liaison* OR advocacy for health OR mediation OR enabling OR empowerment)

We included studies with a trial, quantitative, observational, and longitudinal study design.

We restricted the search to peer-reviewed literature published in English in scientific journals. We did not consider grey literature. No time- or country-related limitations were applied.

Studies have been included and appraised based on relevance and rigor.

Exclusion criteria regarded interventions with only qualitative outcomes or those targeting the paediatric population.

### Search strategy

We searched the following databases: Medline/PubMed, Embase, CINAHL, Scopus, Web of Science, and Social Care Online. The database search was performed from inception to June 30th, 2021. The search strategy is shown in **Table 1**. We analysed the references of identified articles and relevant reviews. We also searched Google Scholar for other potentially relevant studies.

### Article selection and data extraction

Three reviewers (CDA, LS, GA) independently determined the study's eligibility by first reading its title and, if it appeared relevant, the abstract. Finally, they reviewed the full text. Eligible studies were designated “thick” for studies rich in evidence on all relevant elements of CMOs, or as “thin” for studies having only sparse data. Any disagreement was solved through consultation with a fourth reviewer (LG).

Study characteristics were extracted into a table to provide a descriptive overview of the interventions based on the context, mechanism, outcome configuration. We extracted the following information:

- Study details: authors, year of publication, journal, country of intervention delivery, the underlying definition of PHC, study aims, study design, participant characteristics.
- Context: aims of the intervention, type and area of intervention, setting, number of components, contextual factors.
- Mechanism: author-identified mechanisms describing how the intervention influenced outcomes, whether the intervention worked for or not.
- Outcomes: Methods to evaluate success or lack of success of interventions and contextual factors.
- Additional study/information/researcher comments.

### Data synthesis and CMOs identification

The studies were analysed using a realist CMO configuration.

We extracted data from each of the included studies and put them into a table. We sought out data explaining what caused an outcome, through which mechanism, and under which context. The key CMOs in all selected studies were mainly identified from the results sections of the articles. The data extraction process was iterative, with repeated discussion among the research team on the initial programme theories. Throughout the process, they were developed further to reflect the evidence from the included studies. Disagreements were solved through group discussions. Finally, we examined our data extraction table to detect patterns across the selected studies. We followed an interpretative approach to analyse how our data compared with our initial programme theories and modified them accordingly.

Data synthesis was undertaken independently by two researchers (CDA, LS) who met up regularly with the research team to discuss their ongoing analysis.

### Risk of bias

The RAMESES quality assessment training materials and reporting guidelines were followed to assess the included studies' risk of bias. CD and LS appraised studies based on relevance by determining the extent to which a study could contribute to CMO theory building and rigor, which affects the validity of the evidence. Disagreements were resolved through discussion with the research team.

## RESULTS

**Figure 1:**
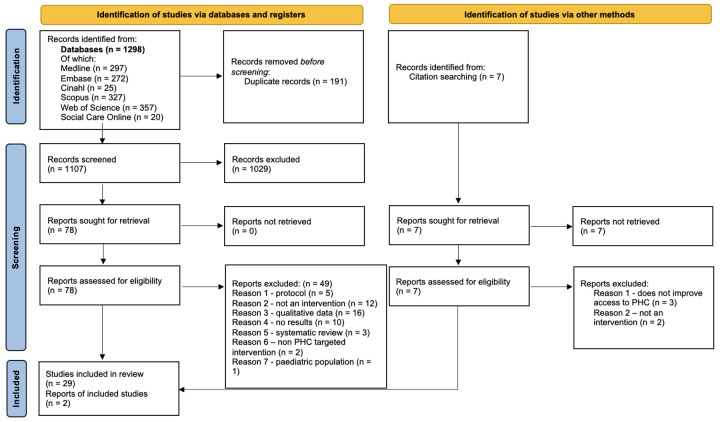
PRISMA 2020 Statement Flow Diagram.

31 studies were included, with 26 categorized as thick and five as thin. A PRISMA 2020 flow diagram [47] for the synthesis process is provided in **Figure 1**.

Of the 31 selected articles, 21 were settled in high-income countries, six in middle-income and upper-middle income countries, and four in low-income countries. Eight studies were described by CMO 1, eight by CMO 2, three by CMO 3. Seven were described by CMO 1 and CMO 3, three by CMO 1 and CMO 2, and two by CMO 2 and CMO 3.

Most interventions were targeted at vulnerable groups. Specifically, 18 interventions addressed vulnerable groups, eight address ethnic groups, and eight address the general population.

Regarding the area of intervention, 21 studies addressed prevention, including screening, 13 addressed treatment, four addressed follow-up, 11 addressed general primary care (see **Table 2**).

**Table 2:** Included studies’ characteristics

Thick/thin		Study design	Income			Country	Population			Area of intervention				CMO			Outcome	
**Thick**	**Thin**	**High**	**Middle**	**Low**		**General population**	**Vulnerable**	**Ethnic minorities**	**Prevention & screening**	**Treatment**	**Follow-up**	**General**	**1**	**2**	**3**	**Positive**	**Negative**
Franz et al. [2020]. Community-based outreach associated with increased health utilization among Navajo individuals living with diabetes: a matched cohort study [48]																		
		Observational cohort study	Y			United States			Y		Y	Y	Y	Y		Y	Y	
Kósa et al. [2020]. Health mediators as members of multidisciplinary group practice: lessons learned from a primary health care model in Hungary [49]																		
Y		Quantitative analysis	Y			Hungary			Y	Y				Y			Y	
Silva-Tinoco et al. [2020]. Role of social and other determinants of health in the effect of a multicomponent integrated care strategy on type 2 diabetes mellitus [50]																		
Y		Before-and-after design		Y		Mexico		Y		Y						Y	Y	
Vieira-Meyer et al. [2020]. Variation in primary health services after implementation of quality improvement policy in Brazil [51]																		
	Y	Observational study		Y		Brazil	Y						Y		Y			Y
Baghirov et al. [2019] Achieving UHC in Samoa through revitalizing PHC and reinvigorating the role of village women groups [52]																		
Y		Commentary of intervention		Y		Samoa	Y			Y	Y			Y			Y	
Lawrence et al. [2019]. Facilitating equitable prevention and management of gout for Maori in Northland, New Zealand, through a collaborative primary care approach [53]																		
Y		Open evaluation based on data collected from patients enrolled in the programme	Y			New Zealand			Y	Y	Y			Y		Y	Y	
Lomonaco-Haycraft et al. [2019]. Integrated perinatal mental health care: a national model of perinatal primary care in vulnerable populations [54]																		
Y		Intervention evaluation	Y			United States		Y		Y	Y					Y	Y	
Mercer et al. [2019]. Effectiveness of Community-Links Practitioners in Areas of High Socioeconomic Deprivation [55]																		
Y		A quasi-experimental cluster-randomized controlled trial	Y			Scotland		Y		Y			Y	Y			Y	
Andrade et al. [2018]. Brazil's Family Health Strategy: Factors associated with programme uptake and coverage expansion over 15 y [1998-2012] [56]																		
	Y	Intervention evaluation		Y		Brazil	Y						Y		Y		Y	Y
Durovni et al. [2018]. The impact of the Brazilian Family Health Strategy and the conditional cash transfer on tuberculosis treatment outcomes in Rio de Janeiro: an individual-level analysis of secondary data [57]																		
	Y	Individual-level analysis of secondary data		Y		Brazil		Y		Y	Y				Y			Y
Gabrielli et al. [2018]. Cervical cancer prevention in Senegal: An International Cooperation Project Report [58]																		
	Y	Intervention - cervical cancer screening programme			Y	Senegal	Y			Y					Y	Y		Y
Hodgins et al. [2018]. The effectiveness of Dental Health Support Workers at linking families with primary care dental practices: a population-wide data linkage cohort study [59]																		
Y		Population-wide data linkage cohort study	Y			Scotland	Y			Y	Y		Y	Y			Y	
Hylviu et al. [2018]. Saving women's lives from cervical cancer: Promoting a cost-effective cervical cancer screening tool in Rural Albania [60]																		
Y		Intervention evaluation		Y		Albania		Y		Y					Y	Y	Y	
Gourley et al. [2017]. Scotland's National Links Worker Programme: mitigating negative impacts of social determinants of health through community connected general practice [61]																		
Y		Intervention evaluation	Y			Scotland		Y		Y			Y	Y			Y	
Lofters et al. [2017]. Lay health educators within primary care practices to improve cancer screening uptake for South Asian patients: challenges in quality improvement [62]																		
Y		Pilot study	Y			Canada			Y	Y				Y				Y
Shavit et al. [2017]. Transitions Clinic Network: Challenges and Lessons in Primary Care for People Released from Prison [63]																		
Y		Through TCN data, assessed the impact of early engagement in primary care and referral from correctional systems to TCN on the use of acute care and recidivism	Y			United States		Y		Y	Y	Y			Y		Y	
Spitzer-Shohat et al. [2017]. Reducing inequity in primary care clinics treating low socioeconomic Jewish and Arab populations in Israel [64]																		
Y		Evaluation of an intervention for inequity-reduction + semi structured interviews	Y			Israel		Y					Y	Y		Y	Y	
Swift [2017]. People powered primary care: learning from Halton [65]																		
Y		Intervention evaluation	Y			England	Y						Y	Y	Y		Y	
Woringer et al. [2017]. Evaluation of community provision of a preventive cardiovascular programme - the National Health Service Health Check in reaching the under-served groups by primary care in England: cross-sectional observational study [66]																		
Y		Cross-sectional observational study	Y			England		Y	Y	Y					Y		Y	
Bhatta and Liabsuetrakul [2016]. Social self-value intervention for empowerment of HIV infected people using antiretroviral treatment: a randomized controlled trial [67]																		
Y		An open-label randomized controlled trial			Y	Nepal		Y			Y		Y		Y		Y	
Brothers et al. [2016]. Young Women Living with HIV: Outcomes from a Targeted Secondary Prevention Empowerment Pilot Trial [68]																		
Y		Behavioral intervention	Y			United States		Y		Y						Y	Y	
Kane et al. [2016]. Improving diabetes care and outcomes with community health workers [69]																		
Y		Mixed methods	Y			United States		Y		Y	Y	Y		Y		Y	Y	
Percac-Lima et al. [2016]. Patient Navigation for Comprehensive Cancer Screening in High-Risk Patients Using a Population-Based Health Information Technology System A Randomized Clinical Trial [70]																		
Y		Randomized clinical trial	Y			United States		Y	Y	Y				Y			Y	
Berkowitz et al. [2015]. Building Equity Improvement into Quality Improvement: Reducing Socioeconomic Disparities in Colorectal Cancer Screening as Part of Population Health Management [71]																		
Y		Interrupted time series analysis before and after a population management intervention	Y			United States		Y		Y				Y	Y		Y	
Percac-Lima et al. [2015]. Patient Navigation to Improve Follow-Up of Abnormal Mammograms Among Disadvantaged Women [72]																		
Y		Evaluation of the impact of a PN programme on follow-up after an abnormal mammogram	Y			United States		Y		Y		Y		Y		Y	Y	
Reeve et al. [2015]. Community participation in health service reform: The development of an innovative remote Aboriginal primary health care service [73]																		
Y		Descriptive mixed-method study	Y			Australia			Y	Y	Y			Y	Y		Y	
Percac-Lima et al. [2014]. The Longitudinal Impact of Patient Navigation on Equity in Colorectal Cancer Screening in a Large Primary Care Network [74]																		
Y		Culturally tailored CRC screening PN programme	Y			United States		Y	Y	Y				Y		Y	Y	
Grasdal and Monstad [2011]. Inequity in the use of physician services in Norway before and after introducing patient lists in primary care [75]																		
Y		Measure horizontal inequity by concentration indices and investigate changes in inequity over time	Y			Norway	Y						Y		Y		Y	
Celletti et al. [2010]. Can the deployment of community health workers for the delivery of HIV services represent an effective and sustainable response to health workforce shortages? Results of a multi-country study [76]																		
Y		Multi-country study			Y	Brazil, Ethiopia, Malawi, Namibia, Uganda		Y			Y		Y	Y			Y	
Sarli et al. [2010]. Training program for community health workers in remote areas in Senegal. First experience [77]																		
	Y	Training programme and post-intervention evaluation			Y	Senegal	Y			Y	Y			Y		Y		Y
Rubenstein et al. [1996]. Evaluation of the VA's pilot program in institutional reorganization toward primary and ambulatory care .1. Changes in process and outcomes of care [78]																		
Y		Intervention with surveys to randomly selected males in 2 phases, practice-based and visit-based	Y			United States		Y			Y				Y		Y	Y
**Total**																		
**26**	**5**		**21**	**6**	**4**		**8**	**18**	**8**	**22**	**13**	**4**	**11**	**18**	**13**	**12**	**26**	**7**
**Thick**	**Thin**		**High**	**Middle**	**Low**		**General population**	**Vulnerable**	**Ethnic minorities**	**Prevention & screening**	**Treatment**	**Follow-up**	**General**	**1**	**2**	**3**	**Positive**	**Negative**
**Thick/Thin**			**Income**				**Population**			**Area of intervention**			**CMO**			**Outcome**		

CMOC – context-mechanism-outcome configuration

The programme theories for each mechanism were presented below and summarized in **Table 3**. It must be noted that these programme theories were not mutually exclusive, with one context and/or mechanism feeding into another or becoming an outcome of a third. However, they have been separated here for clarity.

**Table 3:** Summary of the three context-mechanism-outcome configurations

CMO title	CMO 1 – Health bridge figures	CMO 2 – Tailoring practices	CMO 3 – Training
**Context**	High income countries: general population [59,65], ethnic minorities [48,49,53,62,70,73,74], fragile and vulnerable [55,61,64,69-72,74].	High-income: general population [65,75], ethnic minorities [66,73], fragile and vulnerable [63,66,71,78].	High income countries: ethnic minorities [48,53], fragile and vulnerable [54,64,68-70,72,74].
	Middle-income and upper-middle-income countries: general population [52].	Middle-income and upper-middle income: general population [51,56], fragile and vulnerable [57,60].	Middle-income countries: fragile and vulnerable [50,60].
	Low-income countries: general population [77], fragile and vulnerable [76].	Low-income countries: general population [58], fragile and vulnerable [67].	Low-income: general population [58,77]
**Mechanism**	To insert an ad hoc bridge figure in the health care services familiar with the contextual culture and the users' needs.	Interventions where the staff was asked to consider the needs of targeted populations and differentiate strategies by the detected conditions.	Specific, ad hoc, and contexts-based training targeting several stakeholders, particularly health bridges.
**Positive outcomes**	Increased access to PHC^2^ services [48,49,53,55,59,61,64,65,70-74,76], favoured health equity and decreased barriers of access [53,59,61,64,65,69,70,72-74], favoured empowerment and engagement of service users [48,49,52,69]	Increased access to PHC services [56,57,60,63,65,66,71,75,78], favoured health equity and decreased barriers of access [56,63,65-67,75], favoured empowerment and engagement of service users [60,66]	Increased access to PHC services [48,53,54,60,72], favoured health equity and decreased barriers of access [53,54,64,69,72,74], favoured empowerment and engagement of service users 48,50,60,68,69]
**Negative outcomes**	Did not increase access to PHC services or unevenly raised access [62,77]	Did not increase access to PHC services or unevenly increased access [51,56-58]	Did not increase access to PHC services [58,77]
**n of articles**	18/31	13/31	12/31

CMOC – context-mechanism-outcome configuration, PHC – primary health care

Before moving to the CMOs, we must present some underlying definitions, which we outlined in **Table 4**.

**Table 4:** Working definitions of main concepts from the realist synthesis

Vulnerable – Vulnerability:	**In this study, we referred to groups experiencing “vulnerability” or being “vulnerable” to express that some groups within societies are exposed to contextual conditions that place them more at risk than the rest of the population [** **79** **]. This follows a Determinants of Health approach, recognizing that daily life's structural determinants and conditions led to health inequalities and inequities between and within countries [** **80** **]. We focused on groups whose demographic, geographic, economic, and/or cultural characteristics may impede or compromise their access to PHC^1^ services.**
**Ethnic group:**	An ethnic minority is a group of people who differ in race or color or national, religious, or cultural origin from the dominant group — often the majority population — of the country in which they live.
**Equity:**	Following Whitehead [81]: “Equity in health implies that ideally, everyone should have a fair opportunity to attain their full health potential. More pragmatically, none should be disadvantaged from achieving this potential if it can be avoided.” For this study, equity in health is the absence of systematic disparities in health between groups with different levels of underlying social advantage/disadvantage. Inequalities in health care systematically expose people who are already vulnerable to further disadvantages concerning their health.
**Access to health:**	For this study, access to health is the opportunity to identify health care needs, seek health care services, reach, obtain or use health care services, and have a demand for services fulfilled [7].

PHC – primary health care

### CMO 1: Culturally sensitive ad hoc bridge figure (n = 18)

The mechanism, addressed by 18 papers [48,49,52,53,55,59,61,62,64,65,69-74,76,77], required an ad hoc bridge figure in the health service familiar with the contextual culture and the users’ needs.

Ad hoc figures included community health workers, health mediators, health representatives, health navigators, and village workers. We also noted that this intervention could have been performed by strengthening the capacities of figures already existing in the team. Generally, these figures received specific training, and they usually came from their communities. They knew the specific health needs and culture for bridging the gap between individuals and communities and favoured access to appropriate PHC.

Health bridge figures performed different tasks and respond to different needs. For example, they linked individuals or groups (families, chronic patients) to appropriate health services [48,49,59,61], they supported patients with specific health needs (chronic patients, HIV patients) with treatment and follow-up [69,76], they facilitated communication between patients and services by speaking in their native language [62,72], and they collaborated with the existing health teams to favour health and cultural understanding [64,65].

Except for three studies [57,76,77], this intervention was primarily implemented in high-income countries, under the direction or the support of the national or local health system.

Most of the interventions targeted vulnerable and ethnic groups and were specifically designed to meet those groups’ health and cultural needs.

The introduction of ad hoc health bridge figures and/or the strengthening capacities of figures already existing in the team increased access to PHC services [48,49,53,55,59,61,64,65,70-74], fostered health equity and decreased barriers of access [53,59,61,64,65,69,70,72-74], and favoured empowerment and engagement of service users [48,49,52,69].

By inserting ad hoc bridge figures into the system, several barriers decreased. For example, some studies [59,61,70,72,74] represented cases of reduced barriers in terms of accessibility and acceptability. Three studies [70,72,74] also concerned reduced barriers of accommodation and availability.

All studies discussing this mechanism reported positive outcomes, except for two studies [62,77] which noted that the intervention was unsuccessful and/or it unevenly increased access to PHC. Specifically, one study [62] reported that the intervention was ineffective and too time-consuming. In contrast, according to the second study [77], the intervention was not sustainable in the middle- and long-term. It was not promoted by the local health care system but by an external organization. Therefore, it was not understood by the local population.

The most reported area of intervention addressed prevention, including screening (n = 17), treatment (n = 8), general primary care (n = 7), and follow-up (n = 3).

### CMO 2: Tailoring staff’s practices to the needs of specified populations (n = 13)

This mechanism has been addressed by 13 studies [51,56-58,60,63,65-67,71,73,75,78].

It consisted of interventions where the staff was asked to consider the needs of targeted populations and differentiate strategies following the detected needs.

For example, a study [66] described the dispatch of health staff to communities with the aim of raising awareness of cardiovascular disease prevention in a single consultation without pre-arranged appointments.

In a study on reducing disparities in colorectal cancer screening [71], authors required health staff to integrate information technology services within their practices to facilitate contact with patients and increase screening rates. In contrast, another study [75] introduced a patient list system for general practitioners to improve the stability of the patients-doctors relationship and ensure equity in the use of health services.

One study [63] addressed the creation of a consortium of PHC clinics to support chronically ill prisoners who were recently released, while another study [78] regarded the re-modulation of staff activities of an academic Veteran Affairs medical centre towards PHC.

This intervention was primarily implemented in high-income countries [63,65,66,71,73,75] and mainly targeted vulnerable groups and ethnic ones. Four studies were set in middle and upper-middle-income countries [51,56,57,60], while two were in low-income countries [58,67]. Most actions were directly promoted and implemented by the national or local health care system.

The mechanism allowed health care systems to increase access to PHC services [56,57,60,63,65,66,71,75,78], foster health care equity, and decrease barriers to access [56,63,65-67,75]. Specifically, those barriers pertained to the following dimensions: affordability and availability [56,57,60], accessibility [78], and accommodation [66]. The mechanism also favoured empowerment and engagement of service users [60,66].

All studies discussing this mechanism reported positive outcomes, except for four studies [51,56-58] in which authors noted that the intervention was unsuccessful and/or unevenly increased access to PHC.

Three studies [51,56,57] reported ineffective interventions: they were designed for a general target and not for a specific one. Hence, they did not meet tailored needs, while the fourth study [58] reported an intervention that did not fulfil terms of local economic sustainability.

The most reported area of intervention was prevention, including screening (n = 8), treatment (n = 5), general primary care (n = 5), and follow-up (n = 1).

### CMO 3: Training as the means for staff reskilling (n = 12)

12 studies addressed this mechanism [48,50,53,54,58,60,64,68,69,72,74,77].

The mechanism consisted of specific and context-based training targeting several stakeholders. Central to this intervention was training for health care bridge figures, since they were explicitly trained before performing their duties to cope with the health and social needs of the specific groups they intended to serve [48,53,69,72,74,77]. Nonetheless, training was also designed for other health professionals [54,58,60,64]. In addition, training involving users, particularly chronic patients and communities, was also reported, and was aimed at educating them to perform self-care activities [50,60,68].

Except for four studies [50,58,60,77], this intervention was mainly implemented in high-income countries, with most actions designed to meet vulnerable groups' health and cultural needs.

Specific, needs-tailored and context-based training increased access to PHC services [48,53,54,60,72], fostered health equity and decreased barriers of access [53,54,64,69,72,74], and favoured empowerment and engagement of service users [48,50,60,68,69].

All studies discussing this mechanism reported positive outcomes, except for two studies [58,77]. One study [77] was unsustainable, as it was not promoted by the local health care system but by an external organization. Hence, it was too distant from the needs and understanding of the local community. A second study [58] reported an intervention that did not fulfil terms of local economic sustainability.

The most reported area of intervention was prevention, including screening (n = 12), treatment (n = 5), follow-up (n = 3), and general primary health care (n = 2).

## DISCUSSION

This review aimed to collect and discuss interventions improving access to PHC services to achieve UHC and understand the reasons for the selected interventions' success or lack thereof. Consistent with scientific literature [19,25-29,82], the underpinning theory that guided our analysis is that, for an intervention to be successful, it must consider the context and its contextual factors. Intervention, mechanism, and context are highly intertwined, with all three interacting and influencing each other [29,83].

It was significant that five studies included in the synthesis reported only outcomes of implementing PHC interventions without explaining how or why they occurred. Similarly, some discussed the contexts that influenced the intervention but failed to explain how these conditions and other contextual factors interacted with mechanisms to produce specific outcomes.

Through this systematic review and realist synthesis, we could identify 3 CMO configurations:

- CMO 1: Culturally sensitive ad hoc bridge figures.
- CMO 2: Tailoring staff’s practices to the needs of specified populations.
- CMO 3: Training as the means for staff reskilling.

It is crucial to note that the CMOs were not mutually exclusive, with one context and/or mechanism feeding into another or becoming an outcome of a third. Also, it is worth noting that many studies fell into more than one CMO, with seven studies [48,53,64,69,72,74,77] in CMO 1 and CMO 3, three studies [65,71,73] being encompassed by CMO 1 and CMO 2, and two studies [58,60] described by both CMO 2 and CMO 3. Eight studies [49,52,55,59,61,62,70,76] were only described by CMO 1, eight studies [51,56,57,63,66,67,78] were only described by CMO 2, and three studies [50,54,68] were only described by CMO 3.

From the studies analysed in this review, the most reported intervention was the one described by CMO 1 (n = 18). Healthcare bridges or figures, an umbrella term embracing a variety of selected community health workers, often trained and working in the communities from which they come, increase access to PHC services as they respond to local societal and cultural norms and customs, ensuring community acceptance and ownership. Scientific literature is rich in studies confirming that health bridges can successfully increase access to PHC, especially for vulnerable groups [84-87]. Further, health bridges can be used in response to acute shortages of health workers [88]. CMO 1 is also the most represented in the literature regarding contextual factors for successful and unsuccessful interventions.

Mechanisms encompassed by CMO 2 and CMO 3 are defined in scientific literature as potentially successful interventions for increasing access to PHC. Authors [89,90] described interventions focused on entrusting specific tasks or re-organizing the existing PHC system. In contrast, authors such as Laverack [91] and Caudill et al. [92] reported the importance of empowering health professionals and users through ad hoc training developed upon their specific needs.

Most reported interventions of all CMOs took place in high-income countries and were mainly targeted at vulnerable groups, including ethnic ones. To implement successful and sustainable interventions, it is necessary to have financial and organizational resources. High-income countries have more economical and organizational resources available and, therefore, a greater possibility of developing sustainable interventions. On the other hand, middle- and low-income countries have less economic and organizational resources and consequently a lower opportunity of implementing successful and sustainable interventions. The risk is to start interventions that cannot be continued because of a lack of resources, as was done in the case of the studies by Sarli et al. [77] and by Gabrielli et al. [58], both set in Senegal. Consistently, interventions being supported or implemented by the country’s health organization or in partnership with it have more chances to be successful and sustainable, while interventions fostered by external stakeholders, such as non-governmental organizations, have fewer chances of success, as in the case of the aforementioned studies [58,77].

Regarding the area of intervention, notwithstanding the mechanism nor the country’s level of income, most of the reported studies addressed prevention or/and general PHC actions. This result is not surprising given that both are pivotal pillars of PHC and are strongly fostered by the World Health Organisation (WHO) [2].

Following our theoretical perspective and assumptions, context and contextual factors played a fundamental role in determining the success of an intervention [93]. Regardless of the mechanism, all successful interventions considered the context and its contextual elements by tailoring actions to their target's specific needs and peculiarities, confirming our underpinning theory. These interventions increased access to PHC, raised equity, and reduced health barriers by being so context specific. For example, two studies [59,61] encompassed by CMO 1, through the introduction of the ad hoc health bridge figures in the system, managed to increase access and equity by reducing barriers relating to the dimensions of accessibility and acceptability. On the contrary, interventions that did not consider the context and the contextual factors and generalized their target were not successful, for example, three studies [51,56,57] whose interventions were addressed to the general population and not to a specific group.

The culturally sensitive interventions we discussed involved trained health care workers who can guarantee equity in social care access and promote “empowerment of people, families, and communities so that they can take control of their health and health care” [13]. All the interventions targeting health care services’ acceptability and social determinants, and cultural barriers were strongly context-dependent, like the CMOs we defined.

It should also be noted that those interventions can be profitably combined with other ones for addressing UHC. Investments proved to improve PHC services’ availability for UHC [94], which also benefits from integrating public and private services [95]. Contextual factors are still to be taken into account when developing a fair and sustainable health financing system, because to “ensure that the mechanisms chosen are aligned with country-specific economic, institutional, and cultural characteristics, policymakers need to determine which measures constitute the best or most acceptable means in their specific countries” [95]. Improving capacity and infrastructure, including the workforce, as we have described, especially at the frontline, means strengthening essential PHC and public health functions and moving closer to achieving UHC [13,34].

Scientific literature shows that community-based interventions successfully improve health care access, use, and outcomes when integrated with facility-based services [12]. Community involvement is the cornerstone for developing local, equitable, and integrated PHC, with community members regarded not as passive recipients, but as active leaders in their health [96]. As it emerged from the review, it is fundamental to actively involve the communities by organizing and implementing interventions that specifically answer to their needs by inserting ad hoc culturally sensitive bridge figures (CMO 1), by tailoring staff’s practices to the needs of specified populations (CMO 2), and by training as the means for staff reskilling (CMO 3).

This reasoning is strongly connected with the theme of social determinants of health. Improving PHC needs a multisectoral approach that addresses social (physical and structural) determinants of health [97]. To improve population health, health care equity needs to become a priority in the health sector, and measures to reduce disparities must be integrated into health programmes and services [98]. Interventions that take a holistic approach to health, such as women-focused poverty reduction programmes that include government and civil society institutions, have decreased child mortality and increased equity [99]. Similarly, interventions focused on education and empowerment [50,52], or interventions focused on decreasing barriers [62], paired with the introduction of lay health workers speaking in the language of the minority group, resulted in increased access, changed behaviours, and increased equity.

### Review limitations

The generalizability of the results is limited by the small number of studies identified in the review. Nonetheless, the breath of CMOs, given the diversity of countries where actions were implemented in terms of income levels and health care and cultural settings, can provide valuable indications for the global debate around UHC. Other limitations of the review should be noted. We did not include grey literature, which could amplify data, especially concerning negative results or outcomes. Finally, the heterogeneity of the included studies’ designs prevented us from comparing their results in a quantitative/statistical manner.

## CONCLUSIONS

This review provides policymakers with practical indications for designing potentially successful staff-based interventions to increase PHC access for achieving UHC.

Staff-based interventions come with investments, so middle- and high-income countries may benefit from the implementation of such interventions. In this review, low-income countries are under-represented in the included articles. Nonetheless, more research concerning intervention on PHC access in these settings is desirable. In addition, further research om how evidence in this field influences and is taken into account by policies to achieve UHC should be conducted [100].

The realist synthesis confirms that for such an intervention to be successful, the context and contextual factors regarding the needs of the group it intends to target should be considered. Further, it highlights that for an intervention to have more chances of success and sustainability, it must be supported or implemented by the country's health care organization where the intervention occurs.

## References

[1] United Nations. Transforming our World: The 2030 Agenda for Sustainable Development. 2015. Available: https://sustainabledevelopment.un.org/content/documents/21252030%20Agenda%20for%20Sustainable%20Development%20web.pdf. Accessed: 1 March 2022.

[2] World Health Organization. Primary health care: closing the gap between public health and primary care through integration. Available: https://apps.who.int/iris/rest/bitstreams/1242113/retrieve. Accessed: 1 March 2022.

[3] Wilson D Sheikh A Görgens M Ward K Technology and Universal Health Coverage: Examining the role of digital health. J Glob Health. 2021;11:16006. 10.7189/jogh.11.16006 34912559 PMC8645240

[4] The Lancet. Reinstating universal health coverage on the global agenda. Lancet. 2021;398:2051. 10.1016/S0140-6736(21)02742-2 34863337

[5] Follow-up to the high-level meetings of the United Nations General Assembly on health-related issues. Universal health coverage: moving together to build a healthier world. Report by the Director-General. Available: https://apps.who.int/gb/ebwha/pdf_files/EB146/B146_6-en.pdf. Accessed: 1 March 2022.

[6] World Health Organization. Universal Health Coverage. 2021. Available: https://www.who.int/news-room/fact-sheets/detail/universal-health-coverage-(uhc). Accessed: 1 March 2022.

[7] Levesque J-F Harris MF Russell G Patient-centred access to health care: conceptualising access at the interface of health systems and populations. Int J Equity Health. 2013;12:18. 10.1186/1475-9276-12-18 23496984 PMC3610159

[8] Penchansky R Thomas JW The concept of access: definition and relationship to consumer satisfaction. Med Care. 1981;19:127-40. . 10.1097/00005650-198102000-00001 7206846

[9] Peters DH Garg A Bloom G Walker DG Brieger WR Hafizur Rahman M Poverty and Access to Health Care in Developing Countries. Ann N Y Acad Sci. 2008;1136:161-71. 10.1196/annals.1425.011 17954679

[10] Kullgren JT McLaughlin CG Beyond Affordability: The Impact of Nonfinancial Barriers on Access for Uninsured Adults in Three Diverse Communities. J Community Health. 2010;35:240-8. 10.1007/s10900-010-9230-0 20127505

[11] Ruano AL Furler J Shi L Interventions in Primary Care and their contributions to improving equity in health. Int J Equity Health. 2015;14:153. . 10.1186/s12939-015-0284-6 26694551 PMC4688918

[12] Sacks E Schleiff M Were M Chowdhury AM Perry HB Communities, universal health coverage and primary health care. Bull World Health Organ. 2020;98:773-80. 10.2471/BLT.20.252445 33177774 PMC7607457

[13] Kluge H Kelley E Barkley S Theodorakis PN Yamamoto N Tsoy A How primary health care can make universal health coverage a reality, ensure healthy lives, and promote wellbeing for all. Lancet. 2018;392:1372-4. . 10.1016/S0140-6736(18)32482-6 30343844

[14] Glass DP Kanter MH Jacobsen SJ Minardi PM The impact of improving access to primary care. J Eval Clin Pract. 2017;23:1451-8. . 10.1111/jep.12821 28984018 PMC5765488

[15] van Weel C Kidd MR Why strengthening primary health care is essential to achieving universal health coverage. CMAJ. 2018;190:E463-6 . 10.1503/cmaj.170784 29661815 PMC5903888

[16] Ghebreyesus TA Fore H Birtanov Y Jakab Z Primary health care for the 21st century, universal health coverage, and the Sustainable Development Goals. Lancet. 2018;392:1371-2. 10.1016/S0140-6736(18)32556-X 30343843

[17] World Health Organization. Primary health care. 2021. Available: https://www.who.int/news-room/fact-sheets/detail/primary-health-care. Accessed: 1 March 2022.

[18] World Health Organization. Primary health care on the road to universal health coverage: 2019 monitoring report. 2019. Available: https://apps.who.int/iris/rest/bitstreams/1255038/retrieve. Accessed: 1 March 2022.

[19] Starfield B Shi L Macinko J Contribution of Primary Care to Health Systems and Health. Milbank Q. 2005;83:457-502. 10.1111/j.1468-0009.2005.00409.x 16202000 PMC2690145

[20] World Health Organization. The world health report 2008: primary health care now more than ever. 2008. Available: https://apps.who.int/iris/handle/10665/43949. Accessed: 1 March 2022.

[21] Langlois EV Barkley S Kelley E Ghaffar A Advancing the science and practice of primary health care as a foundation for universal health coverage: a call for papers. Bull World Health Organ. 2019;97:515. 10.2471/BLT.19.239889

[22] Stigler FL Macinko J Pettigrew LM Kumar R van Weel C No universal health coverage without primary health care. Lancet. 2016;387:1811. 10.1016/S0140-6736(16)30315-4 27203497

[23] Thomas L Parker S Song H Gunatillaka N Russell G Harris M Health service brokerage to improve primary care access for populations experiencing vulnerability or disadvantage: a systematic review and realist synthesis. BMC Health Serv Res. 2019;19:269. 10.1186/s12913-019-4088-z 31035997 PMC6489346

[24] Gulliford M Figueroa-Munoz J Morgan M Hughes D Gibson B Beech R What does ‘access to health care’ mean? J Health Serv Res Policy. 2002;7:186-8. 10.1258/135581902760082517 12171751

[25] Øvretveit J Understanding the conditions for improvement: research to discover which context influences affect improvement success. BMJ Qual Saf. 2011;20:i18. 10.1136/bmjqs.2010.045955 21450764 PMC3066695

[26] Evans JM Grudniewicz A Gray CS Wodchis WP Carswell P Baker GR Organizational Context Matters: A Research Toolkit for Conducting Standardized Case Studies of Integrated Care Initiatives. Int J Integr Care. 2017;17:9. 10.5334/ijic.2502 28970750 PMC5624120

[27] Abuzour AS Lewis PJ Tully MP Factors influencing secondary care pharmacist and nurse independent prescribers’ clinical reasoning: An interprofessional analysis. J Interprof Care. 2018;32:160-8. 10.1080/13561820.2017.1394279 29190157

[28] Ploeg J Wong ST Hassani K Yous M-L Fortin M Kendall C Contextual factors influencing the implementation of innovations in community-based primary health care: the experience of 12 Canadian research teams. Prim Health Care Res Dev. 2019;20:e107. 10.1017/S1463423619000483 32800024 PMC8060818

[29] Coles E Wells M Maxwell M Harris FM Anderson J Gray NM The influence of contextual factors on healthcare quality improvement initiatives: what works, for whom and in what setting? Protocol for a realist review. Syst Rev. 2017;6:168. 10.1186/s13643-017-0566-8 28830572 PMC5568400

[30] Khanassov V Pluye P Descoteaux S Haggerty JL Russell G Gunn J Organizational interventions improving access to community-based primary health care for vulnerable populations: a scoping review. Int J Equity Health. 2016;15:168. 10.1186/s12939-016-0459-9 27724952 PMC5057425

[31] Kruk ME Porignon D Rockers PC Van Lerberghe W The contribution of primary care to health and health systems in low- and middle-income countries: A critical review of major primary care initiatives. Soc Sci Med. 2010;70:904-11. 10.1016/j.socscimed.2009.11.025 20089341

[32] Nnaji CA Wiysonge CS Okeibunor JC Malinga T Adamu AA Tumusiime P Implementation research approaches to promoting universal health coverage in Africa: a scoping review. BMC Health Serv Res. 2021;21:414. 10.1186/s12913-021-06449-6 33941178 PMC8094606

[33] Sanogo NA Fantaye AW Yaya S Universal Health Coverage and Facilitation of Equitable Access to Care in Africa. Front Public Health. 2019;7:102. 10.3389/fpubh.2019.00102 31080792 PMC6497736

[34] Jaca A Malinga T Iwu-Jaja CJ Nnaji CA Okeibunor JC Kamuya D Strengthening the Health System as a Strategy to Achieving a Universal Health Coverage in Underprivileged Communities in Africa: A Scoping Review. Int J Environ Res Public Health. 2022;19:587. 10.3390/ijerph19010587 35010844 PMC8744844

[35] Ifeagwu SC Yang JC Parkes-Ratanshi R Brayne C Health financing for universal health coverage in Sub-Saharan Africa: a systematic review. Glob Health Res Policy. 2021;6:8. 10.1186/s41256-021-00190-7 33641673 PMC7916997

[36] Fusheini A Eyles J Achieving universal health coverage in South Africa through a district health system approach: conflicting ideologies of health care provision. BMC Health Serv Res. 2016;16:558. 10.1186/s12913-016-1797-4 27717353 PMC5054620

[37] Myloneros T Sakellariou D The effectiveness of primary health care reforms in Greece towards achieving universal health coverage: a scoping review. BMC Health Serv Res. 2021;21:628. 10.1186/s12913-021-06678-9 34193124 PMC8247133

[38] Myint C-Y Pavlova M Thein K-N-N Groot W A systematic review of the health-financing mechanisms in the Association of Southeast Asian Nations countries and the People’s Republic of China: Lessons for the move towards universal health coverage. PLoS One. 2019;14:e0217278. 10.1371/journal.pone.0217278 31199815 PMC6568396

[39] Atim C Bhushan I Blecher M Gandham R Rajan V Davén J Health financing reforms for Universal Health Coverage in five emerging economies. J Glob Health. 2021;11:16005. 10.7189/jogh.11.16005 34912558 PMC8645242

[40] Uzochukwu BSC Ughasoro MD Etiaba E Okwuosa C Envuladu E Onwujekwe OE Health care financing in Nigeria: Implications for achieving universal health coverage. Niger J Clin Pract. 2015;18:437-44. 10.4103/1119-3077.154196 25966712

[41] Okungu V Chuma J McIntyre D The cost of free health care for all Kenyans: assessing the financial sustainability of contributory and non-contributory financing mechanisms. Int J Equity Health. 2017;16:39. 10.1186/s12939-017-0535-9 28241826 PMC5327514

[42] Pawson R. Evidence-based policy. A realist perspective. London: SAGE Publications; 2006.

[43] MacDonald M Pauly B Wong G Schick-Makaroff K van Roode T Strosher HW Supporting successful implementation of public health interventions: protocol for a realist synthesis. Syst Rev. 2016;5:54. 10.1186/s13643-016-0229-1 27055820 PMC4823871

[44] Jagosh J Realist Synthesis for Public Health: Building an Ontologically Deep Understanding of How Programs Work, For Whom, and In Which Contexts. Annu Rev Public Health. 2019;40:361-72. 10.1146/annurev-publhealth-031816-044451 30633712

[45] Astbury B Leeuw FL Unpacking Black Boxes: Mechanisms and Theory Building in Evaluation. Am J Eval. 2010;31:363-81. 10.1177/1098214010371972

[46] Wong G Greenhalgh T Westhorp G Buckingham J Pawson R RAMESES publication standards: realist syntheses. BMC Med. 2013;11:21. 10.1186/1741-7015-11-21 23360677 PMC3558331

[47] Page MJ McKenzie JE Bossuyt PM Boutron I Hoffmann TC Mulrow CD The PRISMA 2020 statement: an updated guideline for reporting systematic reviews. BMJ. 2021;372:n71. 10.1136/bmj.n71 33782057 PMC8005924

[48] Franz C Atwood S Orav EJ Curley C Brown C Trevisi L Community-based outreach associated with increased health utilization among Navajo individuals living with diabetes: a matched cohort study. BMC Health Serv Res. 2020;20:460. 10.1186/s12913-020-05231-4 32450874 PMC7247176

[49] Kósa K Katona C Papp M Fürjes G Sándor J Bíró K Health mediators as members of multidisciplinary group practice: lessons learned from a primary health care model programme in Hungary. BMC Fam Pract. 2020;21:19. 10.1186/s12875-020-1092-7 31992209 PMC6988313

[50] Silva-Tinoco R Cuatecontzi-Xochitiotzi T De la Torre-Saldaña V León-García E Serna-Alvarado J Guzmán-Olvera E Role of social and other determinants of health in the effect of a multicomponent integrated care strategy on type 2 diabetes mellitus. Int J Equity Health. 2020;19:75. 10.1186/s12939-020-01188-2 32448267 PMC7245830

[51] Vieira-Meyer APGF Machado M de FAS Gubert FA Morais APP Sampaio PY Saintrain MVL Variation in primary health care services after implementation of quality improvement policy in Brazil. Fam Pract. 2020;37:69-80. 31433473 10.1093/fampra/cmz040

[52] Baghirov R Ah-Ching J Bollars C Achieving UHC in Samoa through Revitalizing PHC and Reinvigorating the Role of Village Women Groups. Health Syst Reform. 2019;5:78-82. 10.1080/23288604.2018.1539062 30924751

[53] Lawrence A Scott S Saparelli F Greville G Miller A Taylor A Facilitating equitable prevention and management of gout for Māori in Northland, New Zealand, through a collaborative primary care approach. J Prim Health Care. 2019;11:117-27. 10.1071/HC18082 32171354

[54] Lomonaco-Haycraft KC Hyer J Tibbits B Grote J Stainback-Tracy K Ulrickson C Integrated perinatal mental health care: a national model of perinatal primary care in vulnerable populations. Prim Health Care Res Dev. 2018;20:e77. 10.1017/S1463423618000348 29911521 PMC6567896

[55] Mercer SW Fitzpatrick B Grant L Chng NR McConnachie A Bakhshi A Effectiveness of Community-Links Practitioners in Areas of High Socioeconomic Deprivation. Ann Fam Med. 2019;17:518-25. 10.1370/afm.2429 31712290 PMC6846279

[56] Andrade MV Coelho AQ Xavier Neto M de Carvalho LR Atun R Castro MC Brazil’s Family Health Strategy: factors associated with programme uptake and coverage expansion over 15 years (1998–2012). Health Policy Plan. 2018;33:368-80. 10.1093/heapol/czx189 29346551

[57] Durovni B Saraceni V Puppin MS Tassinari W Cruz OG Cavalcante S The impact of the Brazilian Family Health Strategy and the conditional cash transfer on tuberculosis treatment outcomes in Rio de Janeiro: an individual-level analysis of secondary data. J Public Health (Oxf). 2018;40:e359-66. 10.1093/pubmed/fdx132 29036661

[58] Gabrielli S Maggioni E Fieschi L Cervical cancer prevention in Senegal: an International Cooperation Project Report. Acta Biomed. 2018;89:29-34. 30038201 10.23750/abm.v89i6-S.7460 PMC6357602

[59] Hodgins F Sherriff A Gnich W Ross AJ Macpherson LMD The effectiveness of Dental Health Support Workers at linking families with primary care dental practices: a population-wide data linkage cohort study. BMC Oral Health. 2018;18:191. 10.1186/s12903-018-0650-z 30463549 PMC6249895

[60] Hylviu B Cekodhima G Rista M Shehu B Vandewiele M Huang Y Saving Women’s Lives From Cervical Cancer: Promoting a Cost-Effective Cervical Cancer Screening Tool in Rural Albania. J Glob Oncol. 2018;4:158s. 10.1200/jgo.18.54400

[61] Gourley C Kelvin M Cawston P Scotland’s National Links Worker Programme: mitigating negative impacts of social determinants of health through community connected general practice. Int J Integr Care. 2017;17:450. 10.5334/ijic.3770

[62] Lofters AK Vahabi M Prakash V Banerjee L Bansal P Goel S Lay health educators within primary care practices to improve cancer screening uptake for South Asian patients: challenges in quality improvement. Patient Prefer Adherence. 2017;11:495-503. 10.2147/PPA.S127147 28331296 PMC5352230

[63] Shavit S Aminawung JA Birnbaum N Greenberg S Berthold T Fishman A Transitions Clinic Network: Challenges and Lessons In Primary Care For People Released From Prison. Health Aff (Millwood). 2017;36:1006-15. 10.1377/hlthaff.2017.0089 28583958

[64] Spitzer-Shohat S Shadmi E Goldfracht M Kay C Hoshen M Balicer RD Reducing inequity in primary care clinics treating low socioeconomic Jewish and Arab populations in Israel. J Public Health (Oxf). 2017;39:395-402. 27165669 10.1093/pubmed/fdw037 PMC5896611

[65] Swift M People powered primary care: learning from Halton. J Integr Care. 2017;25:162-73. 10.1108/JICA-12-2016-0050

[66] Woringer M Cecil E Watt H Chang K Hamid F Khunti K Evaluation of community provision of a preventive cardiovascular programme - the National Health Service Health Check in reaching the under-served groups by primary care in England: cross sectional observational study. BMC Health Serv Res. 2017;17:405. 10.1186/s12913-017-2346-5 28615019 PMC5471843

[67] Bhatta DN Liabsuetrakul T Social self-value intervention for empowerment of HIV infected people using antiretroviral treatment: a randomized controlled trial. BMC Infect Dis. 2016;16:272. 10.1186/s12879-016-1634-8 27287712 PMC4902994

[68] Brothers J Hotton AL Hosek SG Harper GW Fernandez MI Young Women Living with HIV: Outcomes from a Targeted Secondary Prevention Empowerment Pilot Trial. AIDS Patient Care STDS. 2016;30:229-35. 10.1089/apc.2015.0294 27158851 PMC4870604

[69] Kane EP Collinsworth AW Schmidt KL Brown RM Snead CA Barnes SA Improving diabetes care and outcomes with community health workers. Fam Pract. 2016;33:523-8. 10.1093/fampra/cmw055 27418587

[70] Percac-Lima S Ashburner JM Zai AH Chang Y Oo SA Guimaraes E Patient Navigation for Comprehensive Cancer Screening in High-Risk Patients Using a Population-Based Health Information Technology System: A Randomized Clinical Trial. JAMA Intern Med. 2016;176:930-7. 10.1001/jamainternmed.2016.0841 27273602

[71] Berkowitz SA Percac-Lima S Ashburner JM Chang Y Zai AH He W Building Equity Improvement into Quality Improvement: Reducing Socioeconomic Disparities in Colorectal Cancer Screening as Part of Population Health Management. J Gen Intern Med. 2015;30:942-9. 10.1007/s11606-015-3227-4 25678378 PMC4471039

[72] Percac-Lima S Ashburner JM McCarthy AM Piawah S Atlas SJ Patient navigation to improve follow-up of abnormal mammograms among disadvantaged women. J Womens Health (Larchmt). 2015;24:138-43. 10.1089/jwh.2014.4954 25522246 PMC4326264

[73] Reeve C Humphreys J Wakerman J Carroll V Carter M O’Brien T Community participation in health service reform: the development of an innovative remote Aboriginal primary health-care service. Aust J Prim Health. 2015;21:409-16. 10.1071/PY14073 25629591

[74] Percac-Lima S López L Ashburner JM Green AR Atlas SJ The longitudinal impact of patient navigation on equity in colorectal cancer screening in a large primary care network. Cancer. 2014;120:2025-31. 10.1002/cncr.28682 24691564

[75] Grasdal AL Monstad K Inequity in the use of physician services in Norway before and after introducing patient lists in primary care. Int J Equity Health. 2011;10:25. 10.1186/1475-9276-10-25 21676210 PMC3141383

[76] Celletti F Wright A Palen J Frehywot S Markus A Greenberg A Can the deployment of community health workers for the delivery of HIV services represent an effective and sustainable response to health workforce shortages? Results of a multicountry study. AIDS. 2010;24:S45-57. 10.1097/01.aids.0000366082.68321.d6 20023439

[77] Sarli L Enongene E Bulgarelli K Sarli A Renda A Sansebastiano G Training program for community health workers in remote areas in Senegal. First experience. Acta Biomed. 2010;81:54-62. 20857853

[78] Rubenstein LV Yano EM Fink A Lanto AB Simon B Graham M Evaluation of the VA’s Pilot Program in Institutional Reorganization toward Primary and Ambulatory Care: Part I, Changes in process and outcomes of care. Acad Med. 1996;71:772-83. 10.1097/00001888-199607000-00009 9158345

[79] Frohlich KL Potvin L Transcending the Known in Public Health Practice. Am J Public Health. 2008;98:216-21. 10.2105/AJPH.2007.114777 18172133 PMC2376882

[80] Marmot M Friel S Bell R Houweling TAJ Taylor S Closing the gap in a generation: health equity through action on the social determinants of health. Lancet. 2008;372:1661-9. 10.1016/S0140-6736(08)61690-6 18994664

[81] Whitehead M The concepts and principles of equity and health. Health Promot Int. 1991;6:217-28. 10.1093/heapro/6.3.217 1644507

[82] Jagosh J Macaulay AC Pluye P Salsberg J Bush PL Henderson J Uncovering the Benefits of Participatory Research: Implications of a Realist Review for Health Research and Practice. Milbank Q. 2012;90:311-46. 10.1111/j.1468-0009.2012.00665.x 22709390 PMC3460206

[83] Pfadenhauer LM Mozygemba K Gerhardus A Hofmann B Booth A Lysdahl KB Context and implementation: A concept analysis towards conceptual maturity. Z Evid Fortbild Qual Gesundhwes. 2015;109:103-14. 10.1016/j.zefq.2015.01.004 26028447

[84] Vaughan JP Walt G Village Health Workers and Primary Health Care. Trop Doct. 1983;13:105-8. 10.1177/004947558301300304 6349058

[85] Hartzler AL Tuzzio L Hsu C Wagner EH Roles and Functions of Community Health Workers in Primary Care. Ann Fam Med. 2018;16:240. 10.1370/afm.2208 29760028 PMC5951253

[86] Sharma N Harris E Lloyd J Mistry SK Harris M Community health workers involvement in preventative care in primary healthcare: a systematic scoping review. BMJ Open. 2019;9:e031666. 10.1136/bmjopen-2019-031666 31852698 PMC6937114

[87] Santos AF da Rocha HA de Lima ÂM de LD de Abreu DMX Silva ÉA de Araújo LHL Contribution of community health workers to primary health care performance in Brazil. Rev Saude Publica. 2020;54:143. 10.11606/s1518-8787.2020054002327 33331421 PMC7702382

[88] Lehmann U, Sanders Da. Community health workers: What do we know about them? The state of the evidence on programmes, activities, costs and impact on health outcomes of using community health workers. 2007. Available: https://www.who.int/hrh/documents/community_health_workers.pdf. Accessed: 1 March 2022.

[89] Rosser WW Kaperski J Organizing Primary Care for an Integrated System. Healthc Pap. 1999;1:5-21. 10.12927/hcpap.1999.17444 12606855

[90] Ugolini C Leucci AC Nobilio L Bertè G Reorganizing territorial healthcare to avoid inappropriate ED visits: does the spread of Community Health Centres make Walk-in-Clinics redundant? BMC Health Serv Res. 2020;20:807. 10.1186/s12913-020-05648-x 32854697 PMC7453714

[91] Laverack G. Public health: power, empowerment and professional practice. New York, NY, US: Palgrave Macmillan; 2005.

[92] Caudill TS Lofgren R Jennings CD Karpf M Commentary: Health Care Reform and Primary Care: Training Physicians for Tomorrow’s Challenges. Acad Med. 2011;86:158-60. 10.1097/ACM.0b013e3182045f13 21270552

[93] Jacobs B Ir P Bigdeli M Annear PL Van Damme W Addressing access barriers to health services: an analytical framework for selecting appropriate interventions in low-income Asian countries. Health Policy Plan. 2012;27:288-300. 10.1093/heapol/czr038 21565939

[94] Assefa Y Tesfaye D Damme WV Hill PS Effectiveness and sustainability of a diagonal investment approach to strengthen the primary health-care system in Ethiopia. Lancet. 2018;392:1473-81. 10.1016/S0140-6736(18)32215-3 30343861

[95] Phua KH Universal health coverage and public-private participation: towards a new balance? Glob Health J. 2017;1:3-11. 10.1016/S2414-6447(19)30078-8

[96] Sacks E Morrow M Story WT Shelley KD Shanklin D Rahimtoola M Beyond the building blocks: integrating community roles into health systems frameworks to achieve health for all. BMJ Glob Health. 2019;3:e001384. 10.1136/bmjgh-2018-001384 31297243 PMC6591791

[97] Rasanathan K Bennett S Atkins V Beschel R Carrasquilla G Charles J Governing multisectoral action for health in low- and middle-income countries. PLoS Med. 2017;14:e1002285. 10.1371/journal.pmed.1002285 28441387 PMC5404752

[98] Andermann A Taking action on the social determinants of health in clinical practice: a framework for health professionals. CMAJ. 2016;188:E474. 10.1503/cmaj.160177 27503870 PMC5135524

[99] Chowdhury MA, Bhuiya A. Do poverty alleviation programmes reduce inequitities in health? The Bangladesh experience. In: Leon DA, Walt G, editors. Poverty, Inequality, and Health: An International Perspective. Oxford, UK: Oxford University Press; 2009. p. 312–32.

[100] Ssengooba F Ssennyonjo A Rutebemberwa E Musila T Namusoke Kiwanuka S Kemari E Research for universal health coverage: setting priorities for policy and systems research in Uganda. Glob Health Action. 2021;14:1956752. 10.1080/16549716.2021.1956752 34402420 PMC8381970

